# Dual‐Band Metasurface‐Based Structured Light Generations for Futuristic Communication Applications

**DOI:** 10.1002/smsc.202400524

**Published:** 2025-02-03

**Authors:** Muhammad Danial Shafqat, Yujin Park, Nasir Mahmood, Joohoon Kim, Dohyun Kang, Rehan Hafiz, Dongliang Gao, Humberto Cabrera, Muhammad Zubair, Muhammad Qasim Mehmood, Lei Gao, Junsuk Rho

**Affiliations:** ^1^ SZCU‐ITU Joint International MetaCenter for Advanced Photonics & Electronics Information Technology University of the Punjab (ITU) Lahore 54000 Pakistan; ^2^ Department of Electrical Engineering Information Technology University of the Punjab (ITU) Lahore 54000 Pakistan; ^3^ MLab STI Unit The Abdus Salam International Centre for Theoretical Physics 34151 Trieste Italy; ^4^ Department of Mechanical Engineering Pohang University of Science and Technology (POSTECH) Pohang 37673 Republic of Korea; ^5^ School of Optical and Electronic Information Jinagsu Key Laboratory of Biophotonics and Suzhou Key Laboratory of Biophotonics Suzhou City University Suzhou 215104 China; ^6^ SZCU‐ITU Joint International MetaCenter for Advanced Photonics and Electronics Suzhou City University Suzhou 215104 China; ^7^ School of Physical Science and Technology and Jiangsu Key Laboratory of Frontier Material Physics and Devices Soochow University Suzhou 215006 China; ^8^ Department of Chemical Engineering Pohang University of Science and Technology (POSTECH) Pohang 37673 Republic of Korea; ^9^ Department of Electrical Engineering Pohang University of Science and Technology (POSTECH) Pohang 37673 Republic of Korea; ^10^ POSCO‐POSTECH‐RIST Convergence Research Center for Flat Optics and Metaphotonics Pohang 37673 Republic of Korea; ^11^ National Institute of Nanomaterials Technology (NINT) Pohang 37673 Republic of Korea

**Keywords:** broadband, metasurfaces, perfect vortex beams, UV, visible

## Abstract

Structured beams carrying orbital angular momentum carry significant potential for various applications, including optical trapping, manipulations, communications, microscopy, and so on. Among these, perfect vortex (PV) beams are highly attractive due to their immunity to topological charge variations and nondiffracting properties. However, conventional PV beam generation methods typically operate at a single wavelength and rely on bulky components, complicating photonic device integration. To address this, a single‐cell‐driven, dual‐band metasurface platform is experimentally demonstrated to generate nondiffracting PV beams spanning from UV to visible wavelengths (261–405 nm). The proposed metasurfaces, made of rectangular‐shaped silicon nitride nanoantennas, achieve an average transmission efficiency of 65% across dual spectrums. Simulated and experimental results show that the metasurfaces maintain topological charge‐insensitive ring radii. The findings highlight a novel approach for PV beam generations, which can lead to new categories of ultrathin optical devices for diverse applications, including wireless communication, particle trapping, and biomedical imaging.

## Graphical abstract

This study introduces a single‐cell‐driven dual‐band metasurface platform generating nondiffracting perfect vortex beams for Ultraviolet–Visible wavelengths. The metasurfaces, composed of silicon nitride nanoantennas, maintain high transmission efficiency across dual spectrums. Experimental results confirm the design's effectiveness in preserving topological charge‐insensitive ring radii. This approach opens possibilities for ultrathin optical devices in wireless communication, particle manipulation, and biomedical imaging applications.

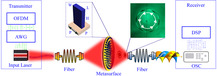

## 1 Introduction

Light structuring refers to the arbitrary control of electromagnetic waves in all their degrees of freedom, including complex amplitude, wavelength, time, polarization, and spatial structuring. Controlling the spatial structures of optical fields allows the generations of numerous specialized light beams, such as focused optical vortex beams, vector beams, Bessel beams, Airy beams, perfect vortex (PV) beams, and spatiotemporal beams. Such structured light beams carrying orbital angular momentum (OAM) have become a significant research area over the past few years and brought numerous breakthroughs to many fields, including micromanipulation,^[^ ^1^ ^]^ quantum information processing,^[^ ^2^ ^]^ optical sensing,^[^ ^3^ ^]^ super‐resolution imaging,^[^ ^4^ ^]^ and OAM‐based optical communications.^[^ ^5^ ^]^ The utilization of OAM‐based mode division multiplexing is instrumental in achieving high data‐carrying capacity resulting from unbounded states and inherent orthogonality, effectively fulfilling the requirement of high‐speed optical communication systems. However, the strong topological charge dependency of the annular intensity distribution of optical vortex beams hindered their real‐life integration, especially coupling into a single air‐core optical fiber.^[^ ^6^ ^]^ On the other hand, the PV beams, generated through a unique combination of multiple optical components, carry OAM and exhibit a topological charge‐insensitive annular intensity profile along the propagation direction.^[^ ^7^ ^]^ The PV beams are renowned for their constant intensity ring radius and evenly distributed OAM density, enabling their applications for communication and nanoparticle trapping and manipulation.^[^ ^7^ ^]^ However, conventional techniques for generating PV beams involve a train of bulky refractive or reflective elements such as Fourier lens, axicon, spatial light modulator, and mirrors, which inevitably increase the system complexity and pose challenges for their integration with on‐chip devices.^[^ ^7^ ^]^

Recently, metasurfaces, an ultrathin version of metamaterials, have shown an unprecedented ability to manipulate the phase, polarization, and complex amplitude of electromagnetic waves with subwavelength spatial resolution and exhibit significant superiority in flexibility and integration‐friendly platform over traditional optical devices.^[^ ^8^ ^]^ Electromagnetic waves’ nanotuning enables to achieve ultracompactness, multifunctionality, higher efficiency, and broadband operational capability and triggers many intriguing applications like flat lensing,^[^ ^9^ ^]^ biomedical imaging and sensing,^[^ ^10^ ^]^ holographic displays,^[^ ^11^ ^]^ 3D integral imaging,^[^ ^12^ ^]^ AI‐driven intelligent surfaces,^[^ ^13^ ^]^ structured light beams generation,^[^ ^14^ ^]^ color filters,^[^ ^15^ ^]^ and optical wireless communication.^[^ ^16^ ^]^ Various novel design techniques for metasurface‐based PV beam generations have recently been proposed, where a train of bulky optical components is efficiently replaced with a single‐layer metadevice.^[^ ^17^ ^]^ However, to the best of our knowledge, including PV beams generating metadevices, most of the pioneering works in metasurfaces for structured light generations have focused on single‐design wavelengths or discrete sets of wavelengths with limited integrated functionalities.^[^ ^18^ ^]^ It is also observed that by intelligently selecting the design parameters of the PV beams generating metasurfaces, a nondiffracting‐type annular intensity distribution can be achieved, rendering them best‐suited candidates for relatively secure information transmission and noninvasive nanoparticle manipulation. Despite the existing research on metasurface‐based structured light generations for futuristic communication applications, the realization of innovative design techniques for broadband multifunctional platforms to generate nondiffracting‐type PV beams could be an apparent technological extension.

This manuscript outlines a numerical investigation and experimental demonstration of a single‐cell‐driven dual‐band metasurface‐based platform for generating nondiffracting‐type PV beams for UV to visible wavelengths (261–405 nm). The platform accomplishes this capability by integrating the phase profiles of the Fourier lens, spiral phase plate, and axicon simultaneously into a single ultrathin structure. The designed metasurfaces, consisting of intelligently optimized dual‐band functional nanoantennas of bandgap‐engineered silicon nitride (Si_3_N_4_) material, exhibit an average transmission efficiency of 65% across the targeted dual bands. The efficacy of the design and resultant metasurfaces has been validated through computational simulations and optical characterization spanning the UV–vis spectral range. For proof of concept, we designed and fabricated several PV beam‐generating metasurfaces with different characteristics, like numerical apertures (NA = 0.4 and 0.8) and topological charges (*l* = 2,4, and 6). The selected characteristics of the PV beams will enable the surpassing of conventional diffraction limits and the demonstration of topological charge immunity of the generated PV beams. We conducted experimental validation using two laser sources: a 360 nm (UV) wavelength and a 405 nm (visible) wavelength. The numerical and optical characterization results confirm that the proposed devices efficiently generate dual‐band PV beams with a uniform annular intensity profile for various topological charges and wavelengths. These recent findings can potentially advance structured light applications in free‐space optical communication, multiplexed communication, optical trapping, optical manipulation, and edge‐enhanced biomedical imaging.

## 2 Theory and Design

**Figure 1:**
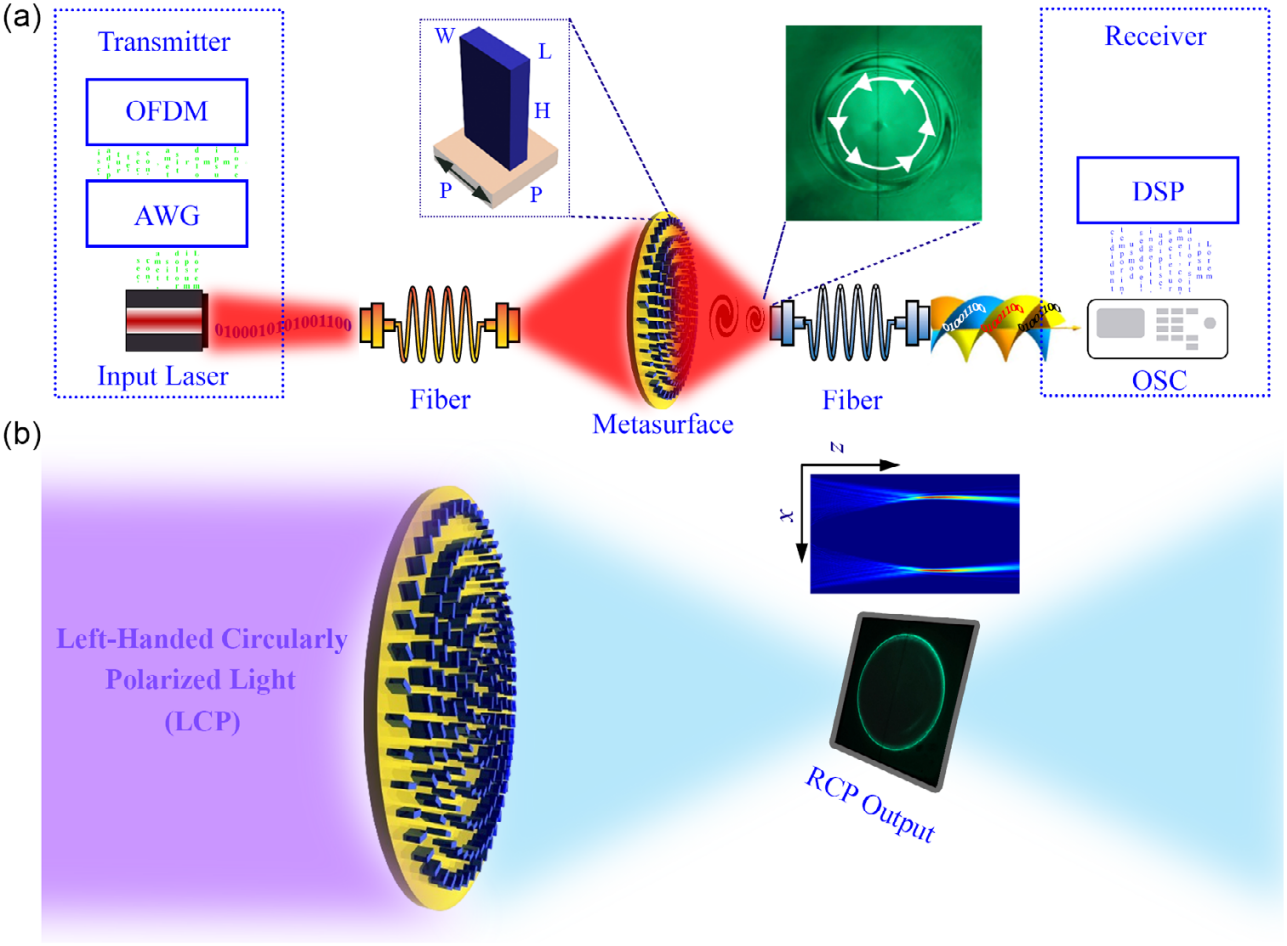
Artistic illustration of the proposed optical wireless communication mechanism and the working principle of a dual‐band functional structured light‐generating metasurface. a) The proposed integration of the designed metasurface within a communication environment. The transmitter section includes orthogonal frequency division multiplexing (OFDM), an arbitrary waveform generator (AWG), and a laser source. On the other hand, the receiver section consists of an oscilloscope (OSC) and a digital signal processing (DSP) system. b) The working principle of the PV beam generating metasurface, where an LCP light illuminates the metasurface from the substrate side, resulting in a long‐propagating topological charge‐insensitive intensity distribution at the output side.

**Figure** **1** depicts an artistic illustration of the proposed optical wireless communication system and the working principle of a dual‐band functional structured light‐generating metasurface. This technology promises to significantly improve future wireless communication's security and information‐carrying capacity. The proposed communication environment can be divided into three segments: the transmission side, the communication path, and the receiving side, as shown in Figure 1a. Information‐carrying signals can be generated by modulating input laser light and transmitting them to the intended receiver. This process involves a communication path with a structured light‐generating metasurface and other necessary components. By utilizing the topological charge‐insensitive ring radius of the generated PV beam, multiple data channels can be efficiently multiplexed and coupled to a single optical fiber. The left inset of Figure 1a presents the fundamental building block of the PV beam‐generating metasurface, consisting of a rectangular‐shaped nanoantenna on a sapphire substrate. The right inset of Figure 1a describes the experimentally recorded intertwined helices indicating the specific topological charge and handedness of the generated PV beam. The operational concept of a PV beam‐generating metasurface is illustrated in Figure 1b, where a left‐hand circularly polarized (LCP) light illuminates the metasurface from the input (left) side, and the resulting diffracted light with opposite helicity is captured and analyzed at the output (right) side. The intensity distribution of the diffracted cross‐polarized light exhibits a unique long‐propagating‐type (inset, Figure 1b) constant ring profile, which remains invariant for different topological charges.

The selected bandgap‐engineered silicon nitride material can be a promising candidate for UV–vis applications due to its complementary metal–oxide–semiconductor compatibility, high energy bandgap (E_g_ = 5.9 eV), and a sizeable transparent window. The bandgap of the Si_3_N_4_ has been modified by managing the flow rate of the silane (SiH_4_) and dinitrogen (N_2_) gas during plasma‐enhanced chemical vapor deposition (PECVD). A 500 nm thick rectangular‐shaped nanoantenna (Figure S1a, Supporting Information) of Si_3_N_4_ is chosen to achieve the desired dual‐band response of the designed metasurfaces. The height of the nanoantenna is selected so that, according to the index waveguide theory, it provides a complete (0–2π) phase coverage. It is well known that the Panchratnam–Berry (PB) phase‐based nanoantennas exhibit inherent broadband performance that deteriorates as the incident wavelength is farther away from the design wavelength. In our design, we also exploited a PB‐phase‐based nanoantenna; however, opposite to the conventional design approach, we optimized the nanoantenna for the desired wavelength bands. The numerically optimized fundamental building block works as a half‐wave plate with a decent average cross‐polarization transmission efficiency over the selected spectrums. A detailed broadband parametric sweep through the FDTD Solution solver is initially applied to the nanoantenna's physical dimensions (length and width). The intensity distribution of the diffracted cross‐polarized light is meticulously analyzed for multiple incident wavelengths, and a unique combination of the length (L = 220 nm) and width (W = 76 nm) of the nanoantenna is chosen that ensures an average transmission efficiency of 65.5%.

Here, it is essential to mention that there are alternative options for the length and width of the nanoantenna for specific incident wavelengths that exhibit relatively higher transmission efficiency than the chosen dimensions. However, selecting such combinations may not meet the single‐cell‐driven, highly efficient broadband design requirement and could lead to a more complex design approach. Figure S1b, Supporting Information, shows the numerical optimization results regarding intensity profiles of cross‐polarized, copolarized light and the average cross‐polarized light within the dual‐wavelength band (261–405 nm). The blue solid line represents the maximum achievable cross‐polarization transmission, the brown solid line represents the copolarized amplitude, and the pink dotted line shows the average cross‐polarized intensity distribution for the chosen dimensions of the nanoantenna. To validate the functionality of the optimized nanoantenna as a broadband half‐wave plate, the nanoantenna is rotated in‐plane from 0–π under selected wavelengths from the interest band. The phase profile of the cross‐polarized light versus in‐plane rotation of the nanoantenna is then studied, as shown in Figure S1c, Supporting Information. With slight deviation, it is confirmed that the optimized nanoantenna provides complete (0–2π) phase coverage for all incident wavelengths (261, 303, 360, and 405 nm), ensuring its functionality as a half‐wave plate for targeted bands.

The designed metadevice integrates the functionalities of multiple optical components (focusing lens, vortex plate, and axicon) into a single‐layer ultrathin structure, significantly reducing the system's overall size. To construct such a device, the phase profiles of these components have been superposed (Equation (1)) and used to dictate the spatial distribution of the nanoantenna, ultimately generating broadband topological charge‐insensitive PV beams with long‐propagating‐type intensity distribution.

(1) $$\varphi_{\text{PV}}(x,y)=\varphi_{\text{spiral\_lens}}(x,y)+\varphi_{\text{axicon}}(x,y)$$

(2) $$\varphi_{\text{spiral\_lens}}(x,y)=-\frac{2\pi }{\lambda_{d}}\left[\sqrt{x^{2}+y^{2}+f^{2}}‐f\right]+l\times \tan^{-1}\left(\frac{y}{x}\right)$$

(3) $$\varphi_{\text{axicon}}\text{(}x\text{, }y)\text{=}-\frac{2\pi }{\lambda_{d}}\times \sqrt{x^{2}+y^{2}}\times \text{NA}$$

Equation (2) presents a combination of a focusing lens and a spiral phase plate, where *f* indicates the focusing distance right above the metasurface and *l* represents the topological charge of the vortex beam. Equation (3) illustrates the phase profile of an axicon with numerical aperture (NA) and designed wavelength *λ*_d_ Conventional design techniques for metasurface‐based PV beam generations have typically resulted in abruptly diverging transmitted light after focusing, which has limited the practical applications of such devices. However, in this article, we have achieved a nondiffracting‐type intensity distribution by intelligently selecting a unique focusing plane for the lens and axicon. This precise adjustment enables diffracted light to constructively interfere along the propagation direction, forming a long‐propagating‐type intensity profile.

## 3 Results and Discussion

**Figure 2:**
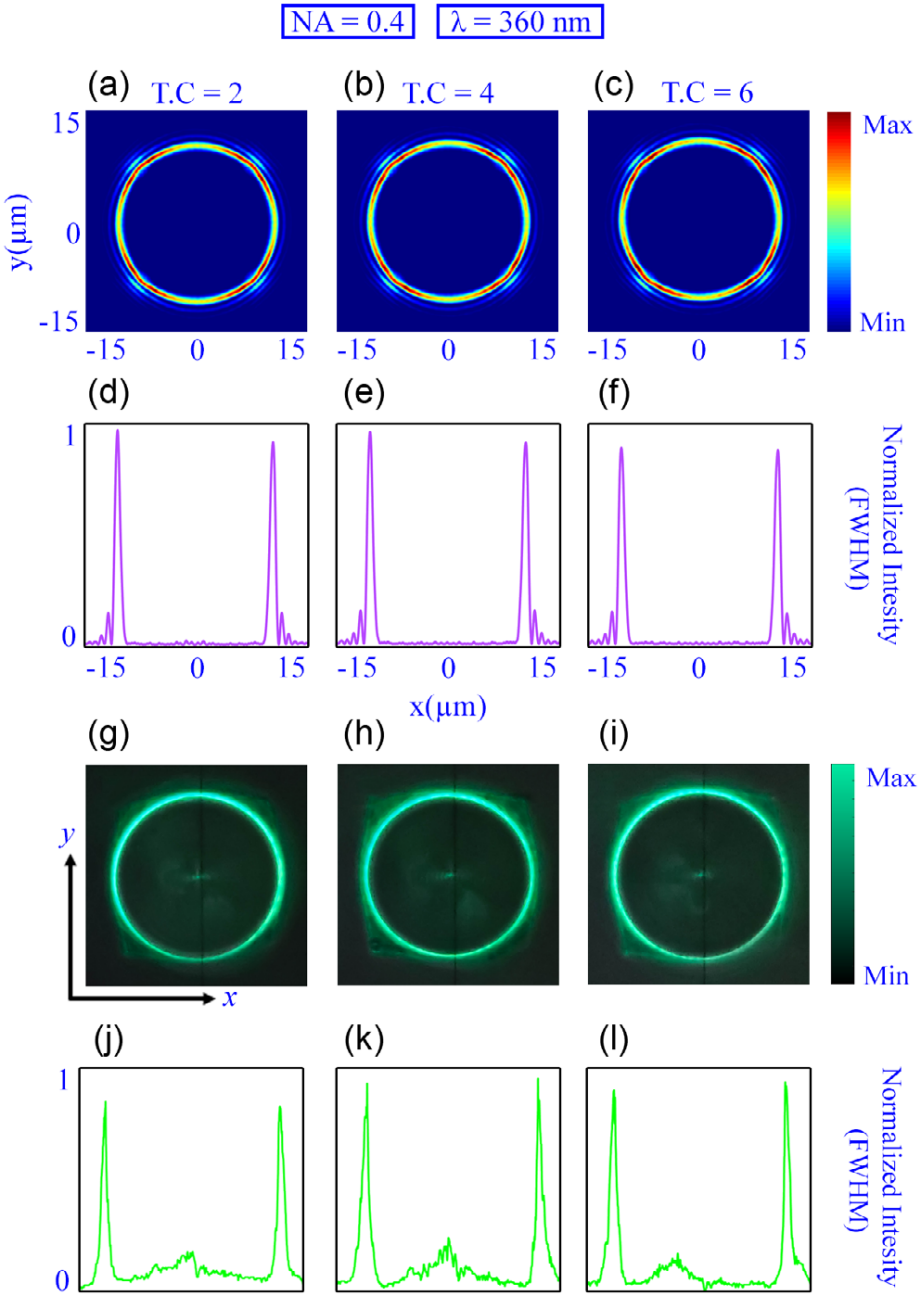
Numerically simulated and optically characterized intensity distribution and FWHM of the generated PV beams for NA = 0.4 and *l* = 2,4, and 6 under 360 nm wavelength light incidence. a–c) The simulated intensity profiles of PV beams. d–f) The FWHM plots derived from numerical simulations indicate a consistent PV beam diameter across all topological charges. g–i) Display the measured intensity profiles of PV beams. j–l) The FWHM plots extracted from the experimental data, confirming a uniform beam diameter for different topological charges, thereby validating the concept of the PV beam generations.

To validate the effectiveness of the proposed technique, we designed several metadevices with different attributes (such as NA and topological charge) of the PV beams. The design parameters for PV beam‐generating metasurfaces are NA = 0.4 and 0.8 and *l* = 2,4, and 6 . Each metadevice underwent thorough numerical analysis and experimental investigation at specific UV–vis spectrum wavelengths. We used the cutting‐edge FDTD Solution 2022 R 1.4 for numerical simulations from Ansys Lumerical Inc., USA. This powerful finite‐difference time‐domain‐based software helps us unlock insights and drive innovation. All numerical simulations are performed for UV–vis wavelengths, specifically 261, 303, 360, and 405 nm. However, two available laser sources from the UV–vis spectrum (360 and 405 nm) are used in optical characterization to illuminate the metasurfaces. **Figure** **2** describes the numerically simulated and optically characterized intensity distribution and corresponding full width at half maximum (FWHM) of the generated PV beams for NA = 0.4 and *l* = 2,4, and 6 under 360 nm incident light. Figure 2a–c presents the simulated surface plot of the intensity distribution at the focusing plane, while Figure 2e–f describes the corresponding FWHM for NA = 0.4 and *l* = 2,4, and 6. Figure 2g–i illustrates the experimental surface plot of the intensity distribution, while Figure 2j–l represents the corresponding FWHM. It is observed that the simulated and experimental intensity distribution exhibit a constant ring radius for different topological charges, which is further verified by the corresponding FWHMs. Similarly, Figure S2, Supporting Information, illustrates the numerical and experimental results of PV beam‐generating metasurfaces for NA = 0.4 and *l* = 2,4, and 6 under 450 nm wavelength light incidence. Figure S2a–c, Supporting Information, describes the simulated intensity profile, and Figure S2e–f, Supporting Information, represents the FWHM for NA = 0.4 and *l* = 2,4, and 6 at the desired focusing plane. Figure S2g–i, Supporting Information, illustrates the experimental intensity distribution, while Figure 2j–l represents the corresponding FWHM. It is examined that the ring radius is invariant for different topological charges, and the recorded intensity distribution remains uniform over the whole ring. Figure S3, Supporting Information, provides the broadband performance of the designed metasurface for NA = 0.4 under the incident wavelengths of 261 and 303 nm. It is verified that, for different incident wavelengths and topological charges, the diameter of the circular intensity pattern remains invariant (Figure S3a–c,g–i, Supporting Information), showing long‐propagating‐type profile along the direction of propagation (Figure S3d–f,j–l, Supporting Information).

**Figure 3:**
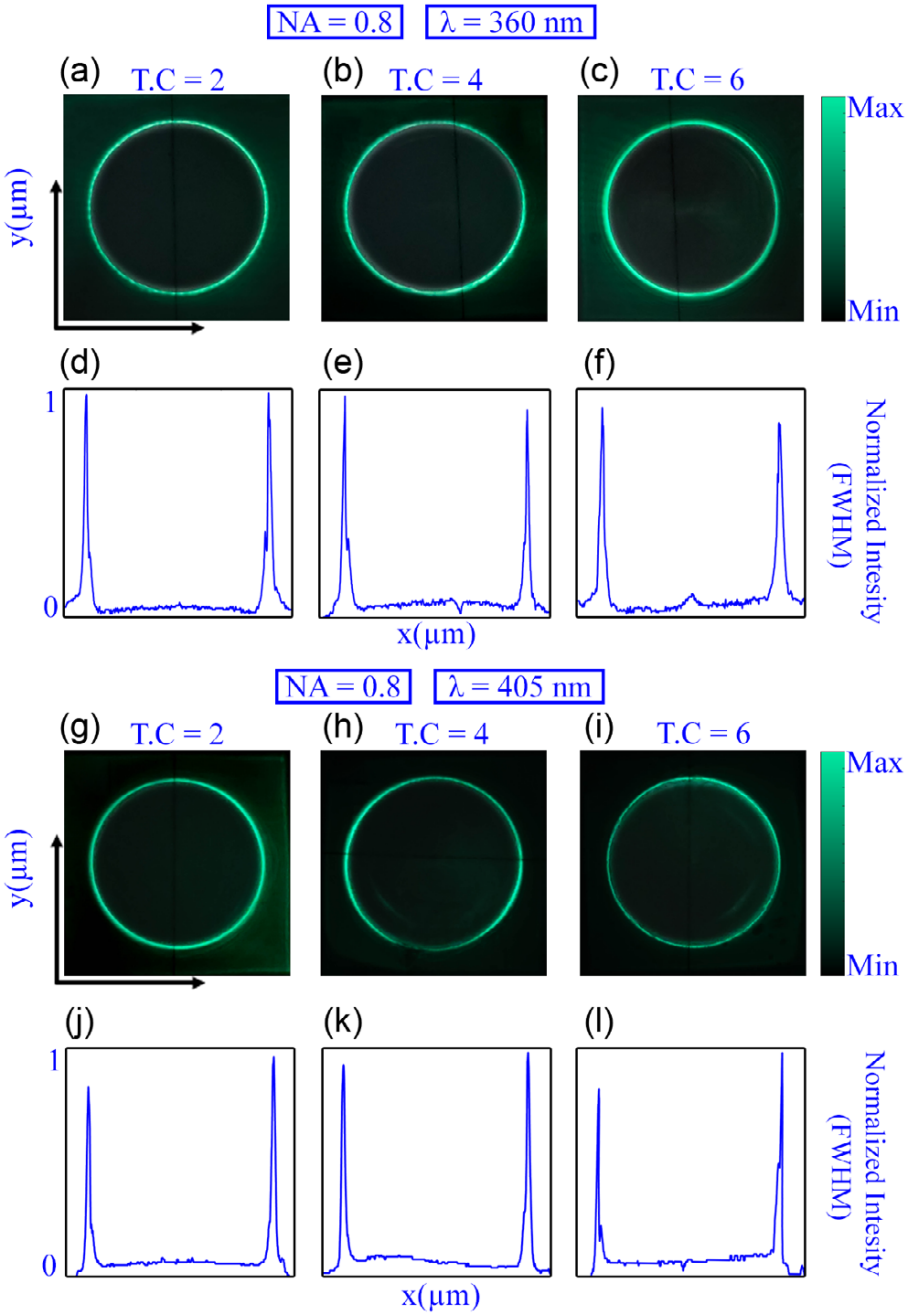
The experimental results of PV beam‐generating metasurfaces for UV–vis wavelengths, 360 and 405 nm, with NA = 0.8. a–c) The measured intensity plots for *l* = 2,4, and 6  illustrate topological charge‐insensitive behavior. d–f) The derived FWHM plots from the experimental data. g–i) The experimental surface plots. j–l) Corresponding FWHM plots for 405 nm wavelength laser source.

To verify the functionality of the proposed design methodology beyond the fundamental limit of conventional diffractive optics of NA = 0.707, we designed several metasurfaces for NA = 0.8 and studied their broadband response. **Figure** **3** describes the experimental results under 360 and 405 nm laser sources for NA = 0.8 and *l* = 2,4, and 6. Figure 3a–c,g–i illustrates the recorded constant intensity profile for different topological charges, while Figure 3d–f,j–l presents the FHWM plots for 360 and 405 nm laser source, respectively. The intensity profile and FWHM demonstrate consistent behavior across UV–vis wavelengths, confirming the effective generations of broadband PV beams with a high NA. Furthermore, the broadband functionality of the proposed design methodology and designed metasurfaces is numerically studied for NA = 0.8 and *l* = 2,4, and 6. Figure S4, Supporting Information, illustrates the simulated profile for NA = 0.8 under UV–vis illumination (261 and 303 nm).

For the numerical investigation of the proposed methodology, we have designed 30 × 30 μm^2^ broadband PV beam‐generating metasurfaces; however, for experimental verification, relatively larger metasurfaces sized at 500 × 500 μm^2^ are fabrication and optical characterization. The fabrication process follows a photolithographic process, in which, first, a 500 nm thick layer of Si_3_N_4_ was deposited on an Al_2_O_3_ substrate using PECVD process while maintaining a flow rate of “1” between silane (SiH_4_) and dinitrogen (N_2_) gas. Then, spin‐coated positive photoresist is exposed using electron beam lithography. After that, the chromium (Cr) layer will be deposited, which acts as an etching mask, and using a drying etching process, Cr patterns are transferred to the Si_3_N_4_ layer. The remaining Cr is wet‐etched, and the metasurface emerges from under it.

**Figure 4:**
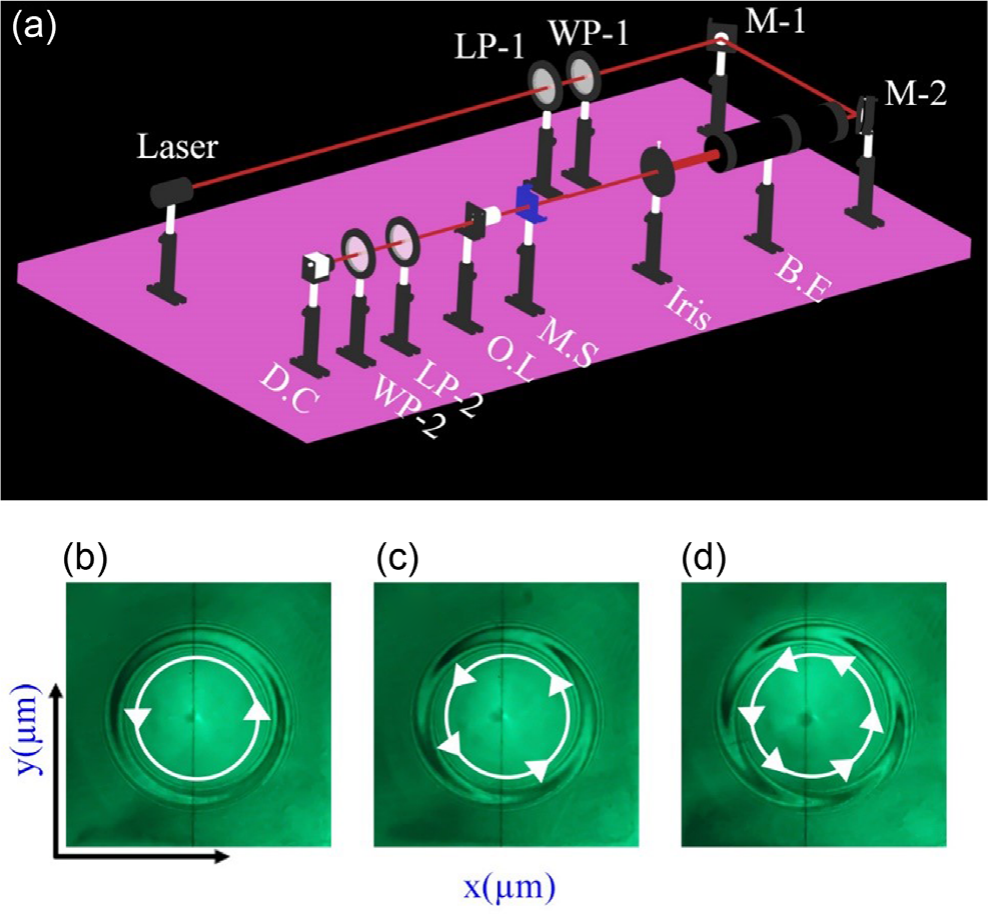
The artistic presentation of custom‐built optical microscopy setup and experimental results for different topological charges. a) Optical setup to characterize the fabricated metasurfaces, which includes an input laser source, a pair of perfectly reflecting mirrors (M‐1, ‐2), a beam expander (BE), an Iris, the metasurface (MS) under test, an objective lens (OL), a pair of linear polarizer and quarter‐wave plate (LP and WP), and UV–vis detector card (DC). b–d) The recorded intertwined helices, indicating the different topological charges (*l* = 2,4, and 6 of the fabricated PV beam‐generating metasurfaces with NA = 0.4.

The fabricated metasurfaces are optically characterized using a custom‐built optical microscopy setup, as shown in **Figure** **4**a. Initially, the input laser light is precisely aligned through a *Z*‐fold configuration using a pair of perfectly reflecting mirrors (M‐1 and ‐2). This configuration provides superior control over the incident light during the optical characterization. In the next step, a beam expander and an iris are placed in the optical path to control the intensity and waist of the laser beam. On the input side, a linear polarizer (LP‐1) and a quarter‐wave plate (WP‐1) are used to transform the randomly polarized input laser light to circularly polarized light of desired handedness. At the same time, another linear polarizer (LP‐2) and a quarter‐wave plate (WP‐2) are configured at the output side to filter out the cross‐polarized light and suppress the unwanted co‐polarized light. The fabricated metadevice under test is then placed immediately after the iris. An objective lens of 50 magnification is used to image and enlarge the output intensity profile, which is finally read by the optical detector. Finally, Figure 4b–d presents the experimentally measured intertwined helices, indicating the different topological charges of the fabricated PV beam‐generating metasurfaces. It is important to note that the intertwined helices or spiral structures illustrated in Figure 4b–d are intrinsic characteristics of the vortex beam‐generating devices, which dictates the integrated topological charge. These spirals become visible at the interface of the metasurface/device. During experimentation, as the objective lens focuses on the metasurface's interface, the intertwined helices are clearly visible and recorded on the observation screen. As the lens is moved further, specific optical phenomena, such as the ring formation of the PV beam, also manifest on the observation screen and are documented. As the topological charge of the PV beam increases, the number of intertwined helices also increases.

## 4 Conclusion

In conclusion, we have numerically investigated and experimentally demonstrated a single‐cell‐driven dual‐band all‐dielectric metasurface‐based platform designed for generating nondiffracting‐type PV beams for UV to visible wavelengths (261–405 nm). The proposed metasurfaces constitute an array of rectangular‐shaped nanoantennas of bandgap‐engineered silicon nitride material and encode the multiplexed phase profiles of several optical elements into a single‐layer ultrathin structure. For proof of concept, we designed, fabricated, and optically characterized multiple metasurfaces for different NAs, like NA = 0.4 and 0.8, and topological charges, like *l* = 2,4, and 6. The selected characteristics of the PV beams will enable the surpassing of conventional diffraction limits and the demonstration of topological charge immunity of the generated PV beams. The numerically simulated and experimental results proved that the proposed design methodology and resultant metasurfaces work perfectly across the UV–vis spectrums, exhibiting an average transmission efficiency of 65% across the dual spectrums. These recent findings can potentially advance the field of structured light applications in various areas, including free‐space optical communication, multiplexed communication, optical trapping, optical manipulation, and edge‐enhanced biomedical imaging.

## Conflict of Interest

The authors declare no conflict of interest.

## Author Contributions

**Muhammad Danial Shafqat**: data curation (lead); formal analysis (lead); investigation (lead); methodology (lead); writing—original draft (lead). **Yujin Park**: data curation (lead); formal analysis (lead); investigation (lead); methodology (lead); writing—original draft (equal). **Nasir Mahmood**: investigation (supporting); methodology (supporting). **Joohoon Kim**: investigation (supporting); methodology (supporting). **Dohyun Kang**: funding acquisition (supporting); investigation (supporting). **Rehan Hafiz**: investigation (supporting); methodology (supporting). **Dongliang Gao**: investigation (supporting); methodology (supporting). **Humberto Cabrera**: investigation (supporting); methodology (supporting). **Muhammad Zubair**: project administration (equal); supervision (equal); validation (equal). **Muhammad Qasim Mehmood**: project administration (equal); supervision (equal); validation (equal); writing—review and editing (equal). **Lei Gao**: funding acquisition (supporting); project administration (equal); supervision (equal); validation (equal); writing—review and editing (equal). **Junsuk Rho**: conceptualization (lead); funding acquisition (lead); project administration (lead); resources (lead); supervision (lead); validation (lead); writing—review and editing (lead). **Muhammad Danial Shafqat, Yujin Park** and **Nasir Mahmood** contributed equally to this work.

## Supporting information

Supplementary Material

## Data Availability

The data that support the findings of this study are available from the corresponding author upon reasonable request.
