# Supplementary material for: Dual‐Band Metasurface‐Based Structured Light Generations for Futuristic Communication Applications
Source: Small Sci. 2025 Feb 3;5(5):2400524. doi: 10.1002/smsc.202400524 (PMC12087768; doi:10.1002/smsc.202400524)
Supplement: Supplementary file 1 — Supplementary Material [file SMSC-5-2400524-s001.pdf]

Supporting Information

Dual-Band Metasurface-Based Structured Light Generations for Futuristic Communication Applications

Muhammad Danial Shafqat,[1,2‡], Yujin Park[3‡], Nasir Mahmood[4,5‡], Joohoon Kim[3], Dohyun Kang[3], Rehan Hafiz[1,2], Dongliang Gao[6], Humberto Cabrera[7], Muhammad Zubair[1,2], Muhammad Qasim Mehmood[1,2*], Lei Gao[4,5*], Junsuk Rho[2,8,9,10,11*]*

Muhammad Danial Shafqat[‡], Muhammad Qasim Mehmood[*], Muhammad Zubair[*], Rehan Hafiz
[1]SZCU-ITU Joint International MetaCenter for Advanced Photonics & Electronics, Information Technology University of the Punjab (ITU), Lahore 54000, Pakistan.
* E-mail: qasim.mehmood@itu.edu.pk, muhammad.zubair@itu.edu.pk

Muhammad Danial Shafqat[‡], Muhammad Qasim Mehmood[*], Muhammad Zubair[*], Rehan Hafiz
[2]Department of Electrical Engineering, Information Technology University of the Punjab (ITU), Lahore 54000, Pakistan.

Yujin Park[‡], Joohoon Kim, Dohyun Kang, Junsuk Rho[*]
[3]Department of Mechanical Engineering, Pohang University of Science and Technology (POSTECH), Pohang 37673, Republic of Korea
* E-mail: jsrho@postech.ac.kr

Nasir Mahmood[‡], Lei Gao[*]
[4]School of Optical and Electronic Information, Suzhou City University, *Jinagsu Key Laboratory of Biophotonics & Suzhou Key Laboratory of Biophotonics*, Suzhou 215104, China
* E-mail: leigao@suda.edu.cn

Nasir Mahmood[‡], Lei Gao[*]
[5]*SZCU-ITU Joint International MetaCenter for Advanced Photonics & Electronics,* Suzhou City University, Suzhou 215104, China

Dongliong Gao
[6]School of Physical Science and Technology & Jiangsu Key Laboratory of Frontier Material Physics and DevicesThin Films, Soochow University, Suzhou 215006, China.

Humberto Cabrera
[7]MLab, STI Unit, The Abdus Salam International Centre for Theoretical Physics, 34151, Trieste, Italy

Junsuk Rho
[8]Department of Chemical Engineering, Pohang University of Science and Technology (POSTECH), Pohang 37673, Republic of Korea

Junsuk Rho
[9]Department of Electrical Engineering, Pohang University of Science and Technology (POSTECH), Pohang 37673, Republic of Korea

Junsuk Rho
[10]National Institute of Nanomaterials Technology (NINT), Pohang 37673, Republic of Korea

Junsuk Rho
[11]POSCO-POSTECH-RIST Convergence Research Center for Flat Optics and Metaphotonics, Pohang 37673, Republic of Korea

[‡] **These authors have contributed equally to this work.**

Keywords: Ultraviolet, Visible, Metasurface, Perfect vortex beam, Broadband

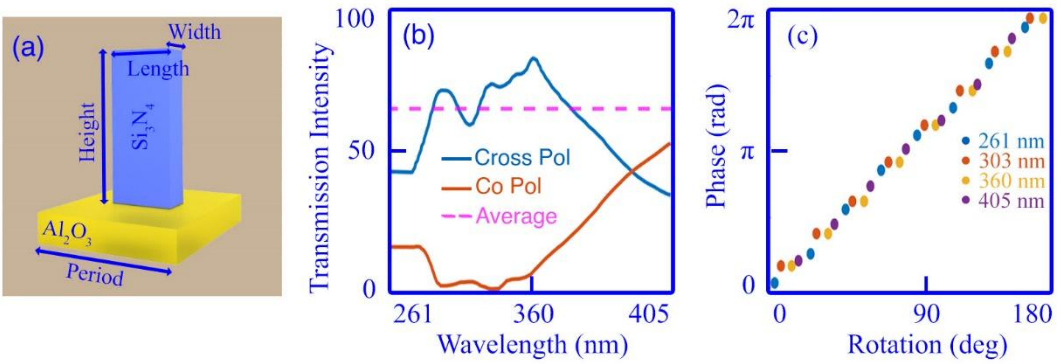

Figure S1. The fundamental building block and its numerical optimization results. (a) The schematic of a $500\ \mathrm{nm}$ tall optimized nanoantenna with length $(\mathrm{L} = 220\ \mathrm{nm})$ and width $(\mathrm{W} = 76\ \mathrm{nm})$, while the period of the building block is $(\mathrm{P} = 290\ \mathrm{nm})$. (b) The transmission intensity profile shows the co-polarization, cross-polarization, and average polarization efficiency achieved after the nanobars were optimized. (c) The analysis of the phase coverage of the nanobars demonstrates complete phase coverage across the selected wavelengths.

Figure S1(a) illustrates the perspective view of the fundamental building block of the proposed broadband non-diffracting-type PV beam-generating metasurfaces. It consists of a rectangular-shaped Si_3N_4 nanoantenna sitting on a sapphire substrate. The simulated results of numerical optimization are presented in Fig. S1(b) and (c), describing the intensity distribution of cross- and co-polarized light vs. incident wavelength and the broadband complete phase coverage.

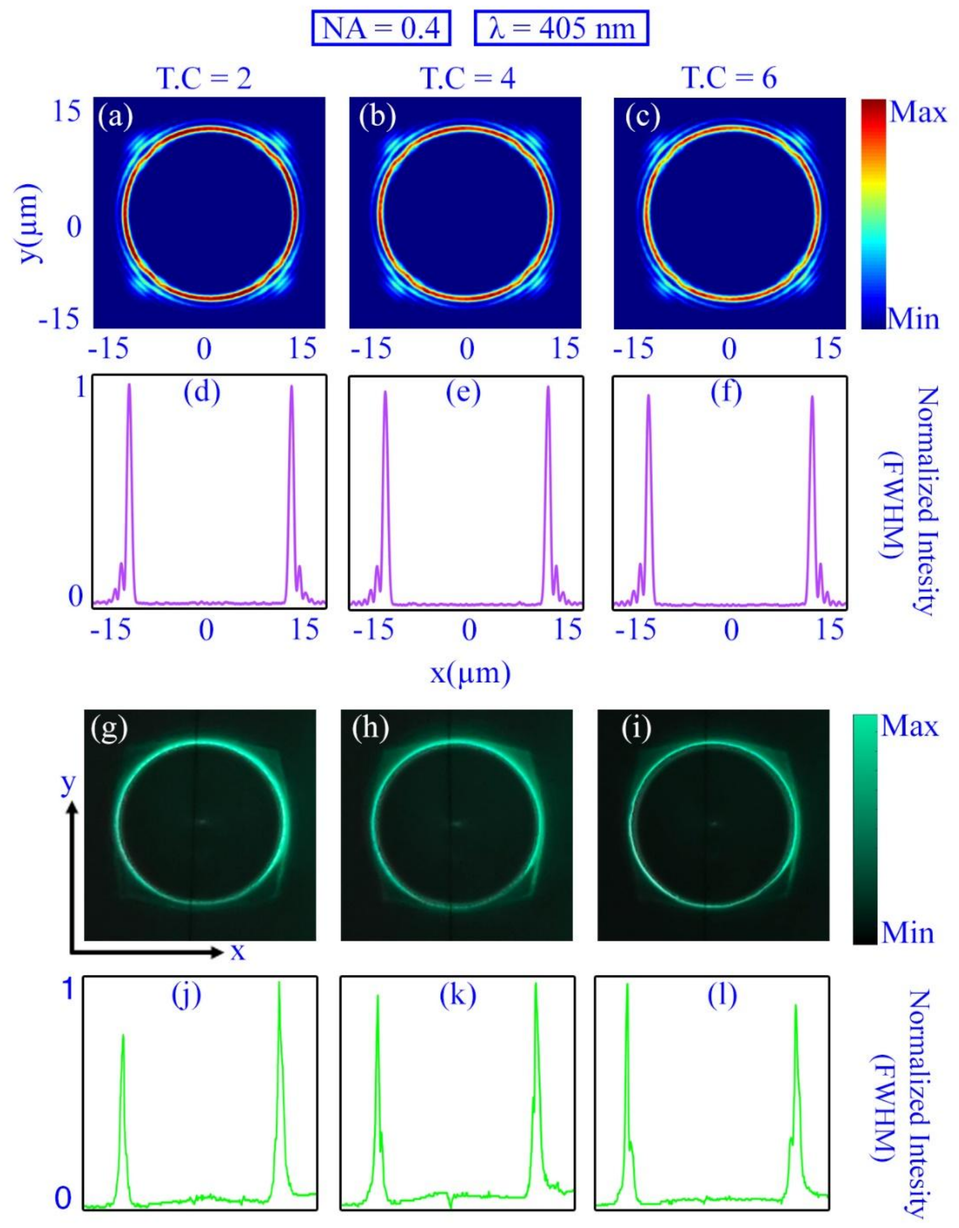

Figure S2. Numerically simulated and optically characterized intensity distribution and FWHM of the generated PV beams for NA $= 0.4$ and $l = 2, 4,$ and 6 under a 405 nm wavelength light incidence. (a-c) The simulated intensity profiles of PV beams. (d-f) The FWHM plots derived from numerical simulations indicate a consistent PV beam diameter across all topological charges. (g-i) Display the measured intensity profiles of PV beams. (j-l) The FWHM plots extracted from the experimental data, confirming a uniform beam diameter for different topological charges, thereby validating the concept of the PV beam generations.

Fig. S2 illustrates the numerical and experimental investigation of the designed metasurfaces for NA $= 0.4$ and $l = 2, 4,$ and 6 under 450 nm wavelength light source. Figure S2(a-c) illustrates the simulated diffracted intensity distribution, and Fig. S2(e-f) represents the full-width at half-maximum (FWHM) for NA $= 0.4$ and $l = 2, 4,$ and 6 at the desired focusing plane. Figure S2(g-i) illustrates the experimental intensity distribution, while Fig. 2(j-l) represents the corresponding FWHM.

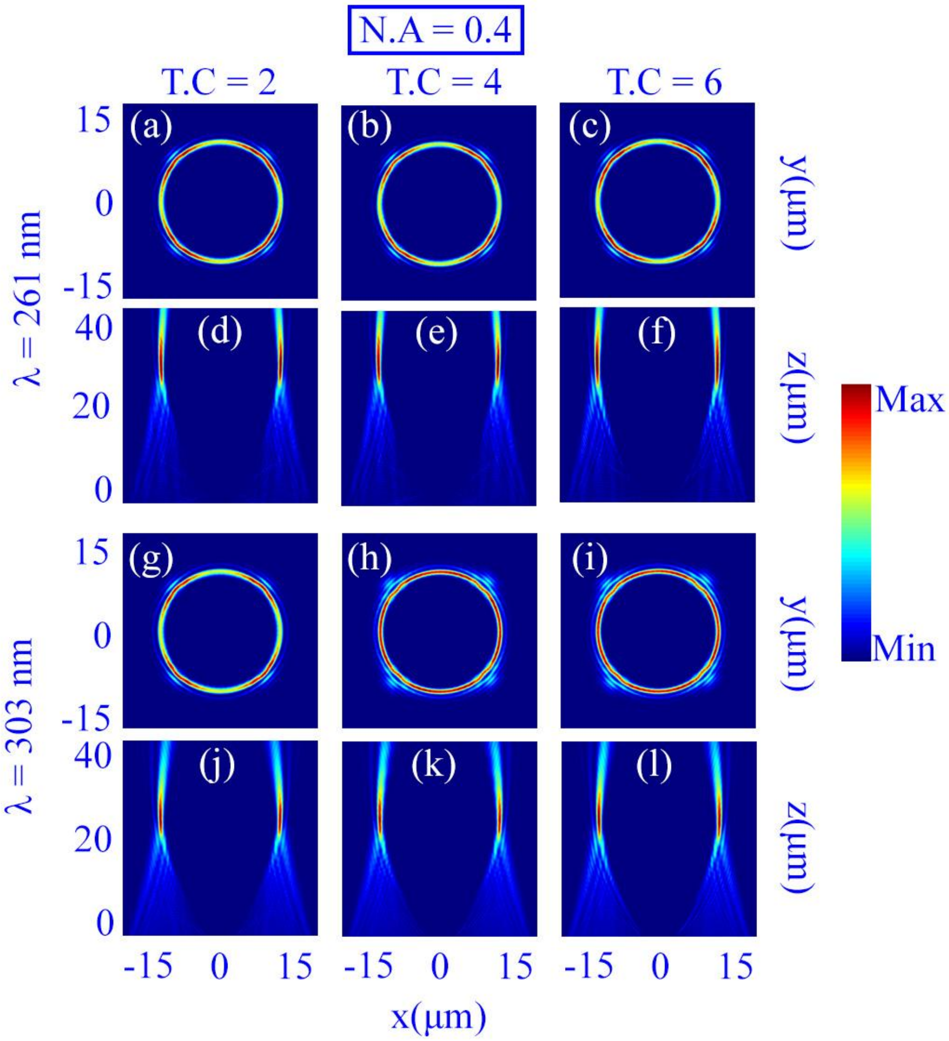

Figure S3. The broadband performance of the designed metasurface for NA = 0.4 under the incident wavelengths of 261 and 303 nm. It is verified that, for different incident wavelengths and topological charges, the diameter of the circular intensity pattern remains invariant (Fig. S3(a-c) and (g-i)), showing long-propagating-type profile along the direction of propagation (Fig. S3(d-f) and (j-l)).

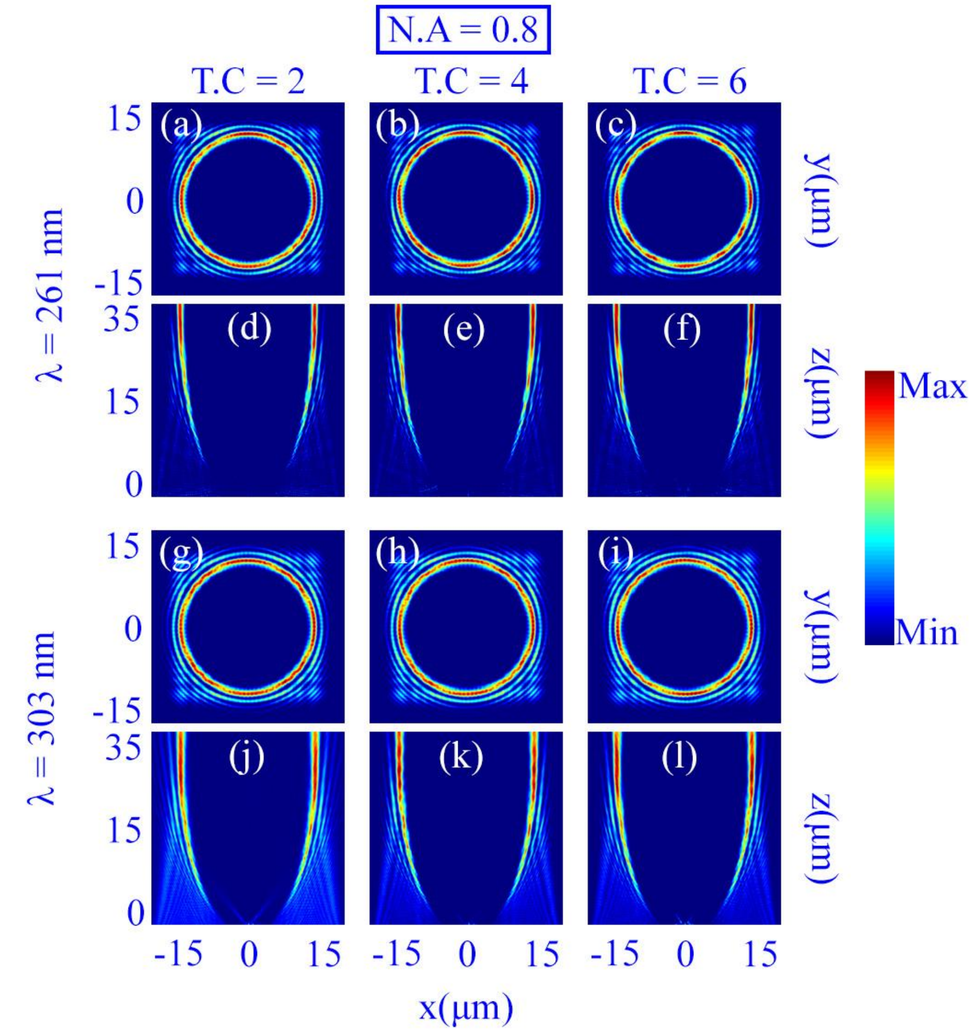

Fig. S4. The broadband performance of the designed metasurface for NA = 0.8 under the incident wavelengths of 261 and 303 nm. It is verified that, for different incident wavelengths and topological charges, the diameter of the circular intensity pattern remains invariant (Fig. S3(a-c) and (g-i)), showing long-propagating-type profile along the direction of propagation (Fig. S3(d-f) and (j-l)).
